# Causal inference from observational data in neurosurgical studies: a mini-review and tutorial

**DOI:** 10.1007/s00701-025-06450-6

**Published:** 2025-02-12

**Authors:** Mingxuan Liu, Xinru Wang, Jin Wee Lee, Bibhas Chakraborty, Nan Liu, Victor Volovici

**Affiliations:** 1 Center for Quantitative Medicine, Duke-NUS Medical School, Singapore, Singapore; 2 Duke-NUS Medical School, Programme in Health Services and Systems Research, Singapore, Singapore; 3 Department of Statistics and Data Science, National University of Singapore, Singapore, Singapore; 4 Department of Biostatistics and Bioinformatics, Duke University, Durham, NC USA; 5 Institute of Data Science, National University of Singapore, Singapore, Singapore; 6 SingHealth AI Office, Singapore Health Services, Singapore, Singapore; 7 Department of Neurosurgery, Erasmus MC Rotterdam, Rotterdam, The Netherlands; 8 Center for Complex Microvascular Surgery, Erasmus MC Rotterdam, Rotterdam, The Netherlands

**Keywords:** Causal inference, Neurosurgical research, Epidemiology

## Abstract

**Background:**

Establishing a causation relationship between treatments and patient outcomes is of essential importance for researchers to guide clinical decision-making with rigorous scientific evidence. Despite the fact that randomized controlled trials are widely regarded as the gold standard for identifying causal relationships, they are not without its generalizability and ethical constraints. Observational studies employing causal inference methods have emerged as a valuable alternative to exploring causal relationships.

**Methods:**

In this tutorial, we provide a succinct yet insightful guide about identifying causal relationships using observational studies, with a specific emphasis on research in the field of neurosurgery.

**Results:**

We first emphasize the importance of clearly defining causal questions and conceptualizing target trial emulation. The limitations of the classic causation framework proposed by Bradford Hill are then discussed. Following this, we introduce one of the modern frameworks of causal inference, which centers around the potential outcome framework and directed acyclic graphs. We present the obstacles presented by confounding and selection bias when attempting to establish causal relationships with observational data within this framework.

**Conclusion:**

To provide a comprehensive overview, we present a summary of efficient causal inference methods that can address these challenges, along with a simulation example to illustrate these techniques.

**Supplementary Information:**

The online version contains supplementary material available at 10.1007/s00701-025-06450-6.

## Introduction

Driven by evidence-based medicine, clinical neurosurgical research has increasingly focused on investigating causation, not just association [10, 42, 53]. Despite the widely-held belief thatRandomized Controlled Trials (RCTs) represent the gold standard for inferring causal effects, recent studies have emphasized their limitations in neurosurgical research, such as ethical issues and high cost. These issues highlight the importance and need for a paradigm shift, to investigate causality from non-randomized and observational data [8, 15, 61, 67]. For instance, studies have explored the causal effects of early surgical interventions for spinal cord injuries [6] and secondary glioblastoma multiforme resection [16] in non-randomized cohorts. A common challenge in these observational studies is to address selection and confounding biases, which can distort the observed causal relationship between treatment and outcome. Causal inference aims to account for these biases and disentangle causation from association [20, 40].

A historical milestone occurred when Sir Austin Bradford Hill [23], in an early effort to formalize an understanding of causal relationships, proposed nine criteria for identifying causal effects (Table 1). Rather than imposing a rigid set of guidelines, Hill’s criteria were intended to serve as a flexible framework to aid in the discovery of causal effects, an intellectual game [17]. However, concerns have been raised about the implausibility, vagueness, and lack of a strict definition of causal effects in Hill’s criteria [17, 20, 56, 69]. These criticisms cast doubt on Hill’s criteria, suggesting that alternatively, more modern approaches could be used.

**Table 1:** Details of the Bradford Hill criteria

Criteria	Definition
Strength	A stronger association between the exposure and the outcome suggests a higher likelihood of a causal relationship between these two factors
Consistency	A consistent association between the exposure and the outcome within multiple studies conducted in different locations, population, and methods suggests a higher likelihood of a causal relationship between these two factors
Specificity	A specific association between the exposure and the outcome, i.e., the association causes only one disease suggests a higher likelihood of a causal relationship between these two factors
Temporality	For a causal relationship to be established, the exposure must occur before the outcome
Biological gradient	A dose–response relationship, e.g., increased level of exposure corresponds to increased incidence of disease, suggests a higher likelihood of a causal relationship between these two factors
Plausibility	For a causal relationship to be established, an association between the exposure and the outcome should be consistent with the current biological and etiological knowledge
Coherence	For a causal relationship to be held, an association between the exposure and the outcome should be consistent with the currently available knowledge in all related domains
Experiment	Experimental manipulation may provide the strongest evidence for a causal relationship between the exposure and the outcome
Analogy	Strong evidence of a causal relationship between the exposure and the outcome indicates a higher likelihood of a causal relationship between a similar exposure and the outcome

Since Hill’s criteria, the field of causal inference has seen numerous developments, largely driven by the potential outcome framework, also known as the Rubin Causal Model [26, 49]. This framework defined causal effects as the expected difference between potential outcomes for each treatment assignment. However, only one potential outcome can exist and be observed for each patient; for example, if a patient undergoes resection, the outcome of non-resection for the same patient under the same conditions (called in epidemiological terms the *counterfactual*) is purely hypothetical [24]. The fundamental challenge of causal inference, from this perspective, is the absence of outcomes for the unreceived treatment [24], which must be rigorously addressed through quantitative techniques. To address this challenge as well as common confounding and selection bias, this paper aims to provide researchers with a guide for applying causal inference techniques in neurosurgery with observational data, thereby enhancing the quality of causality-based decision-making.

## Preparation: identifying the causal question and emulating a trial

To properly employ observational data for causality investigation, it is crucial to identify a specific causal question. Understanding the causal effects of treatments on the targeted outcome can greatly aid clinical decision-making. For instance, neurosurgeons may want to investigate whether early surgery will reduce the risk of long-term complications after spinal cord injury [5, 6], or the causal effects of a second resection on survival probability in recurrent glioblastoma [16]. In addition to treatment effects, researchers may also wish to investigate the causal effects of multiple factors, such as pre-injury, injury-related, and clinical variables in traumatic brain injury [41]. In this case, Pirracchio et al. identified two significant factors, the history of hepatic disease and the history of psychiatric disease, both of which are associated with a poorer functional outcome. [41] Furthermore, causal questions can be extended to uncover causal relationships among closely related factors, [25]. Moreover, exploring effect modification, whereby treatment effects differ across subpopulations, is of paramount importance, especially when anticipating population heterogeneity. The causal questions will further guide the selection of covariates and the determination of causal methods. In this study, we concentrate on estimating treatment effects due to its prevalence in the scientific literature and potential for future studies.

The target trial emulation principle in causal inference is to simulate a hypothetical RCT when an actual RCT is not feasible due to ethical issues, logistical or cost issues [19, 36]. After specifying the clinical question, as in an RCT, defining a targeted trial involves specifying eligibility criteria for participants, treatment definitions, causal estimands, follow-up duration, baseline time point, and outcome of interest. The observational data is then used to emulate the targeted trial by finding eligible individuals, assuming randomization, following them from trial start to end, and conducting an analysis adjusted for confounding and selection bias.

The treatment assignment strategy is crucial, where randomness is virtually impossible in observational studies given that control over treatment assignments is limited [45]. To improve such a situation, causal inference techniques can be utilized to derive desired causal effects. The success of the emulation relies on assessing the plausibility of causal assumptions and examining the sensitivity of the results to potential biases or unmeasured confounders [67].

## Beyond Bradford Hill: new tools for causal inference

The Bradford Hill criteria [22], consisting of nine criteria (Table 1), have been widely employed to evaluate the evidence for causal relationships between a presumed exposure and an outcome of interest [12, 18, 37]. However, advances in biological, etiological, and statistical domains necessitate re-evaluating these criteria to enable causal inferences [13, 17]. As Fedak et al. [17] stated, statistical developments allow investigators to test the strength of association from both the magnitude and statistical significance perspectives. Additionally, the suitability and interpretability of the analytical model employed should be given greater consideration. Despite the Bradford Hill criteria providing a systematic framework, they may fall short in addressing issues such as confounding and selection bias, thereby limiting their applicability in current real-world clinical settings [13] (Table 2).

**Table 2:** Assumptions in the potential outcome framework

Assumption	Formula	Description
Consistency	${Y}_{i}={T}_{i}{Y}_{i}^{\left(1\right)}+\left(1-{T}_{i}\right){Y}_{i}^{\left(0\right)}$	The individual with treatment ${T}_{i}$ has the observed outcome ${Y}_{i}$ equal to the potential outcome under the treatment they received
Exchangeability	${Y}_{i}^{\left(0\right)},{Y}_{i}^{\left(1\right)}\perp {\text{T}}_{\text{i}}$	The treatment assignment does not depend on potential outcomes
Positivity	$0<Pr({T}_{i}=t)<1$ for $t=0, 1$	The probability of assigning to each treatment is higher than zero

Aside from the Bradford Hill criteria, other causal inference frameworks have been proposed, as evidenced in the Olsen and Jensen essay, who argue in favor of a so-called “consequence criterion”, i.e. implementing a vaccine for a deadly disease even in the presence of less-than-ideal evidence may be warranted [38].Shepherd’s criteria can help evaluate if an exposure is teratogenic or not [57]. Also in this case a consequence criterion applies, even if the evidence is limited, if the evidence is correct then the consequences are grave.

In response to the limitations of the Bradford Hill criteria, the *Potential Outcome (PO) framework* [50] and *Directed Acyclic Graphs (DAG)* [34] have emerged as valuable tools for establishing causal relationships. These complementary approaches offer a more comprehensive understanding of causation by visualizing causal pathways and accounting for confounding variables. Although other causal inference methods exist, such as do-calculus [34] and Bayesian decision analysis [14], we outline the PO framework and DAG in the Supplementary Appendix.

## Determine the appropriate causal inference methods

To produce reliable ATE estimates, various methods such as standardization, propensity score matching, targeted maximum likelihood, etc., can be employed, as detailed in this section. These methods aim to establish comparability between the exposure and non-exposure groups, thereby reducing the impact of confounding or other biases. It is important to note that these methods still rely on corresponding assumptions (Table 3) and violating these assumptions can result in biased estimates.

**Table 3:** Summary of causal methods

Method	Assumptions*	Advantages	Limitations
Standardization	• Consistency • Conditional exchangeability • Positivity	• Provides a straightforward estimation of causal effects • Easily interpretable results	• Assumes correct specification of the outcome model and sensitivity to model misspecification • Relies on the availability of all relevant covariates
Propensity score-based method	• Consistency • Conditional exchangeability • Positivity	• Can be used with various statistical techniques (e.g., matching, weighting, stratification) • Identify positivity assumption violations	• Assumes correct specification of the propensity score model and sensitivity to model misspecification
TMLE	• Consistency • Conditional exchangeability • Positivity	• Provides consistent estimates even with misspecified models • Can handle complex exposure scenarios	• Can be computationally intensive

^*^ Causal inference methods generally require the assumptions of no unmeasured confounding and no measurement error in covariates

### Standardization

Standardization involves calculating expected outcomes across strata divided by confounding variables with the total dataset as a reference, assuming the dataset’s distribution is consistent with the actual population [20, 27, 29, 40, 44]. When the number of confounders and their categories are manageable, and the sample size is sufficiently large, non-parametric standardization can be useful. This involves computing mean outcomes in each subgroup defined by the confounders—a form of "within-stratum" adjustment [27]. The overall treatment effect can be obtained using methods such as weighted average based on the frequency of each confounder subgroup or meta-analysis [21]. When there are many confounders or some have multiple levels (or some are continuous variables), parametric standardization using techniques such as regression should be considered [67].

### Propensity score-based methods

Propensity score (S), defined as the conditional probability of assignment to a particular treatment given a group of observed variable, i.e., ${S}_{i}=P\left({T}_{i }\right|{X}_{i})$, is a classical tool to address selection bias [46]. In practice, true propensity scores are rarely known outside of randomized experiments and thus need to be estimated by models such as logistic regression and generalized boosted models [1]. The main applications of propensity score include model adjustment, stratification, weighting, and matching. The propensity score can be directly added to the outcome prediction model to adjust for confounders or to stratify the data for subgroup analysis. The other two directions, weighting and matching, are explained as below.

Propensity score weighting assigns each individual a weight based on their propensity score, and the ATE can be estimated using statistical methods like weighted regression [4]. The idea is to create a "pseudo-population" in which the distribution of covariates is balanced between treated and control groups, mimicking the conditions of a randomized controlled trial [60]. There are two main approaches to constructing weights based on propensity scores: a) inverse probability of treatment weighting (IPTW) weighting [11], where individuals are weighted by $1/S$, if they received treatment, and by $1/(1-S)$, if they did not; b) overlap weighting [62], where individuals are weighted by $1-S$, if they received treatment, and directly by $S$, if they did not. The latter puts more emphasis on individuals with propensity scores close to 0.5, where the treated and control groups overlap the most.

Propensity score matching, as one of the matching methods [58, 59], relies on matching the treated and control units with similar propensity scores. This balances the distribution of confounders between the treatment and control groups, removing the effects of treatment assignment on covariates [51, 52]. To perform matching based on the propensity score, there are several methods such as nearest-neighbor matching [47] and caliper matching [3], which differ in how they prioritize balance between the groups and overall sample size. Nevertheless, in certain cases the unobserved confounders may become imbalanced when matching on observed confounders, which make propensity score matching only as powerful as the dataset and provided confounders.

### Targeted maximum likelihood estimation (TMLE)

Targeted Maximum Likelihood Estimation (TMLE) is a semi-parametric method used in causal inference to estimate causal effects in the presence of confounding variables [63–65]. This method is particularly suitable for complex observational data since it combines the strengths of machine learning and statistical techniques to provide robust and efficient estimates of treatment effects.

TMLE is a doubly robust maximum-likelihood–based approach, where the robustness is achieved by modelling both the treatment assignment mechanism (e.g., propensity scores) and the outcome mechanism (e.g., regression of the outcome on the treatment and covariates) [54]. Initially, these two models are initially separately built. Then a “clever covariate” is constructed to capture the relationship between these two mechanisms, upon which the initial outcome regression model is updated to a targeted version. This method uniquely combines machine learning and logistic regression to create a more robust confounder adjustment.

### Examples in neurosurgical studies

Propensity score matching is a commonly used approach in neurosurgical studies. Balas et al. applied this approach to respectively estimate the causal effects of early surgery on the risk of complications for acute traumatic thoracolumbar spinal cord injury [6] and complete cervical spinal cord injury [5]. Fariña Nuñez et al. [16] also conducted propensity score matching and validated the matching results using t-Distributed Stochastic Neighbor Embedding (t-SNE). Based on the matched cohort, a Cox proportional-hazards regression model with re-resection as a time-dependent covariate was computed [16]. Koenecke et al. [28] applied both propensity score trimming (excluding individuals with extreme propensity values) in combination with IPTW and propensity score matching to investigate the causal effects of Alpha-1 adrenergic receptor (⍺1-AR) antagonists on preventing hyperinflammation and death in patients with acute respiratory distress and pneumonia. TMLE and its extension collaborative TMLE (cTMLE) were applied by Pirracchio et al. [41] to compute the causal importance ranking of variables towards the disability score at three months post-injury.

## Evaluate the strength of the evidence

Despite addressing potential confounding and selection biases through the methods introduced above, the causal estimate may still be inaccurate due to factors such as unmeasured confounders or measurement errors. Therefore, it is essential to consider the limitations and potential sources of bias when interpreting causal conclusions drawn from an observational study. Multiple statistical methods should be conducted to evaluate the robustness of the results. Furthermore, incorporating relevant biological and etiological knowledge, conducting replication studies, and performing sensitivity analysis can provide additional evidence to support or against causal conclusions, ultimately strengthening the validity of the findings.

## Causal inference on observational dataset: a demo

**Fig. 1:**
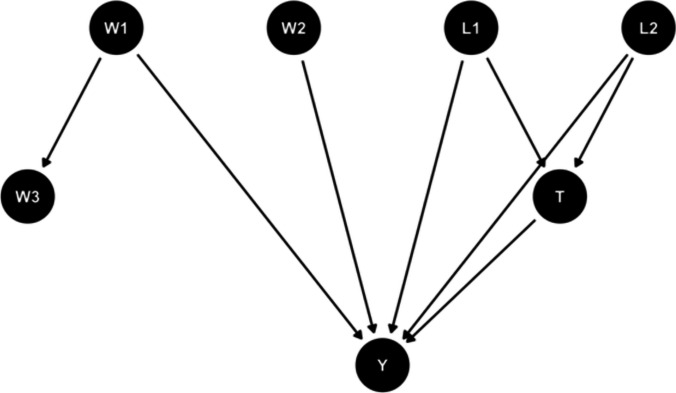
The DAG (directed acyclic graphs) in the setting of simulation. ${L}_{i}$ is the confounder that needs to be adjusted for

To demonstrate the applications of causal inference methods, we designed a simulation setting that satisfied the assumptions of consistency, conditional exchangeability, and positivity. The setting included a binary outcome ($Y$), a binary treatment ($T$), two confounders (binary ${L}_{1}$ and continuous ${L}_{2}$) influencing both the treatment and the outcome, and two outcome-related covariates (${W}_{1}$ and ${W}_{2}$) independent of treatment assignment. When conditioning on ${W}_{1}$, ${W}_{3}$ and $Y$ are independent. Figure 1 illustrates the relationship between variables and the outcome. We adjusted confounders ${L}_{1}$ and ${L}_{2}$, to ensure the causal relationship between treatment $T$ and outcome $Y$, and included covariates ${W}_{1}$ and ${W}_{2}$ for modeling. ${W}_{3}$ was excluded due to its conditional independence from $Y$ in the presence of ${W}_{1}$. It is crucial to approach the selection of covariates and identification of confounding factors with the uttermost caution in practice [66].

**Fig. 2:**
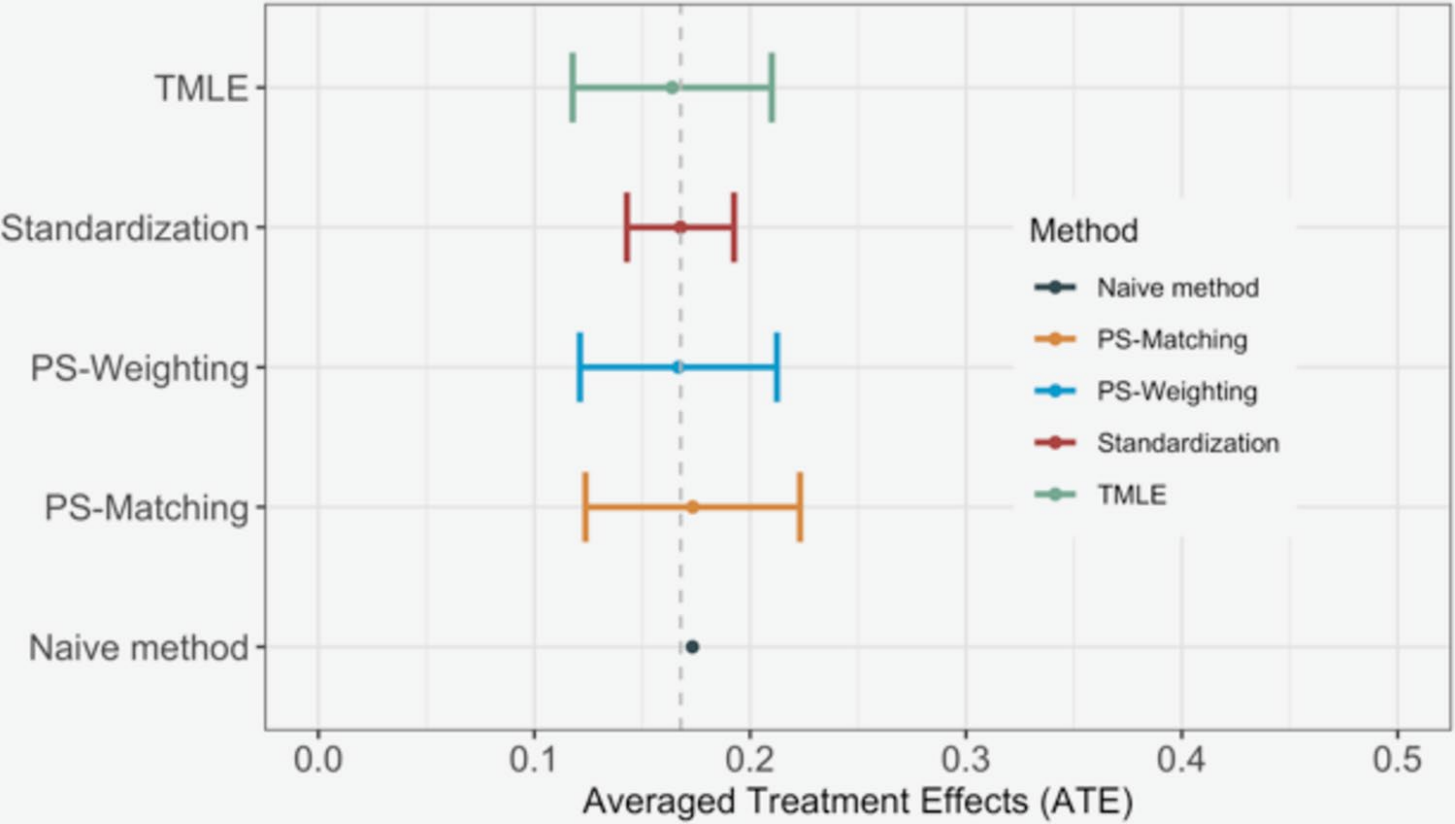
The estimation of ATE (average treatment effects) via different causal inference methods. TMLE: targeted maximum likelihood estimates; PS: propensity score

We applied the aforementioned methods to estimate the ATE in our analysis: 1) standardization, particularly, parametric standardization, as there was a continuous confounder 2) Propensity score weighting via IPTW 3) Propensity score matching via optimal full matching that supports the calculation of ATE 4) TMLE. The baseline method for comparison is the “naïve method”, which directly computed the difference in the average outcomes between two treatment subgroups. The objective was to obtain the ATE of treatment $T$ on outcome $Y$. The implementation codes can be found in the supplementary material. The results for the estimated ATE by each method are presented in Fig. 2. The true ATE in this setting was 0.168. The “naive method” exhibited an overestimation of the ATE, whereas parametric standardization, propensity score-based methods (weighting and matching), and TMLE yielded similar estimates, with standardization providing the most accurate estimate.

## Discussion

In this tutorial, we provide a concise yet insightful guide for researchers to identify causal relationships through observational studies. Researchers are encouraged to begin by clearly defining the specific causal questions they intend to investigate, conceptualizing a hypothetical randomized trial that aligns with their research question, and then designing the observational study with the target trial emulation approach. On top of Bradford Hill’s criteria, we highlighted the potential outcome framework and DAG as one of the fundamental pillars of causal inference and introduced several commonly used causal inference methods. These statistical approaches can address various bias sources, particularly confounding and selection bias, enabling researchers to obtain more robust and reliable causal conclusions to guide the neurosurgical practice.

### Thinking causal inference as grounds before study inception

When utilizing observational data for causality investigation, it is crucial to approach it with the same rigor as conducting an RCT, starting from the study setup phase. The core idea is to make the data from observational studies resemble data from a randomized experiment as closely as possible, as emphasized in Sect. 2.1. Some of the limitations of observational data can be solved by taking a proactive stance from the beginning.

In observational studies, the ambiguity regarding treatment timing can be resolved by accurately recording the timing of treatment decisions. The issue of design and analysis mingling, which involves simultaneous access to covariates, treatment, and outcome, can be mitigated by isolating the outcome from the analysis until the treatment groups are sufficiently balanced [19, 36]. Another common limitation of observational study is the lack of a pre-specified protocol for analysis [2, 9]. This can be addressed by establishing a rigorous protocol in advance and specifying the planned statistical methods and analysis steps. By implementing these proactive strategies, researchers can enhance the validity and reliability of causal inference from observational data, minimizing potential biases and limitations [19, 36].

### Choices along the causal inference process

To draw valid causal conclusions, it is essential to choose appropriate causal inference methods, considering the nature of the data, research questions of interest, and required assumptions of each method. Particularly, in neurosurgical studies with rare diseases and multiple covariates, propensity score-based methods are often preferable to (parametric) standardization [30, 39]. In addition, methods based on propensity score offer advantages in identifying and addressing positivity assumption violations [31, 70]. For example, observations with extreme scores may be excluded from analysis to obtain more efficient estimates. However, discarding data may result in power loss and sample differences from the target population [55]. When researchers have greater confidence in correctly specifying the outcome model rather than the propensity model, standardization can be a superior choice. Conversely, if there is limited information about both the outcome model and the propensity model, TMLE can provide a robust estimation of causal quantities [63–65].

Variable selection in both propensity and outcome models is crucial in causal inference. Researchers should rely on both neurosurgical domain knowledge and statistical considerations to effectively identify potential confounding variables that require adjustment and colliders that should not be adjusted for. It is advised to include all variables believed to be correlated with the outcome in the propensity model, while excluding variables only related to the exposure [55]. For parametric standardization, advanced techniques, such as random forest, Super Learner, and Gradient Boosting Machines (GBM), offer more accurate estimates of potential outcomes by effectively selecting important variables and capturing complex relationships and interactions among them.

Missing data problems also threaten the validity of causal inference. The missing mechanisms, as described by Little and Rubin [33, 48], are related to the degree of exposure and outcome dependency. When missing completely at random (MCAR), i.e., the missingness does not depend either on the exposure or on the outcome, complete-case analysis that simply drops missing values can yield unbiased results. When the missingness depends on the exposure but not the outcome, defined as missing at random (MAR), multiple imputations can provide an unbiased causal estimate that accounts for the uncertainty introduced by imputation. Inverse probability weighting (IPW) can also address the missingness under the MAR assumptions. Furthermore, sensitivity analysis is needed to evaluate the robustness of conclusions under each missing mechanism [32].

### Causal inference in current neurosurgery

Despite being considered in some neurosurgical studies, as mentioned in Sect. 4.4, causal inference remains underutilized in this field. In addition to the investigation of treatment effects, which is the main focus of this study, causal inference can also contribute to identifying risk factors associated with neurosurgical conditions or surgical outcomes, providing researchers with insights about the modifiable risk factors that can be targeted to improve patient outcomes. Furthermore, the mediation analysis [43] in causal inference can be applied to investigate the mechanisms by which neurosurgical treatments or risk factors influence outcomes.

### Future directions

While our primary focus is on estimating the Average Treatment Effect (ATE), it is imperative to acknowledge other estimands of interest. For instance, the Average Treatment Effect on the Treated (ATT) becomes relevant when evaluating the treatment’s impact exclusively on those who received it. Notably, this review focused on fixed treatment comparisons without additional adjustments. However, real clinical practice often involves individualizing and adjusting treatments based on patients’ characteristics and evolving disease status when dealing with relapsing and chronic diseases.

Dynamic treatment regimens (DTRs) are sequences of decision rules that can formalize adaptive disease management plans and guide healthcare providers on which treatment should be given to which subgroup of patients [7]. Although the sequential multiple assignment randomized trial (SMART) serves as the gold standard for constructing optimal DTRs [68], longitudinal observational data can be leveraged in situations where randomization is not feasible, allowing for the evaluations of DTRs at a lower cost. However, when using observational data to estimate treatment effects of each DTR, it is crucial to pay meticulous attention to time-varying confounders, as treatment assignment may vary based on patients’ intermediate disease status and characteristics. Mahar et al. [35] provided a comprehensive overview of relevant statistical methods, such as IPW, G-estimation, Q-learning, etc., for constructing optimal DTRs using observation longitudinal data. Readers can refer to Hernan and Robins [20] for estimating various causal estimands and corresponding statistical techniques in different contexts.

## Conclusion

In this study, we emphasized the critical importance of causation in clinical research and provided a concise guide to identifying causal relationships through observational datasets. We strongly encourage clinical researchers to delve into the field of causal inference and appropriately apply these causal methods to enhance the quality of evidence and improve patient care.

## Supplementary Information

Below is the link to the electronic supplementary material.

Supplementary Material 1 (DOCX 18.0 KB)

## Data Availability

The data is generated and the code will be provided upon written reasonable request to the corresponding author.
