# Supplementary material for: Causal inference from observational data in neurosurgical studies: a mini-review and tutorial
Source: Acta Neurochir (Wien). 2025 Feb 12;167(1):40. doi: 10.1007/s00701-025-06450-6 (PMC11813971; doi:10.1007/s00701-025-06450-6)
Supplement: Supplementary file 1 — Supplementary Material 1 (DOCX 18.0 KB) [file 701_2025_6450_MOESM1_ESM.docx]

- 1. **The potential outcome (PO) framework**

The potential outcomes (PO) framework defines potential outcomes for each patient under different treatment conditions. For each patient $i$ ($i= 1, \ldots, n$, where $n$ is the sample size), the binary treatment indicator $T_{i}$ denotes under-exposure ($T_{i}=1$) and or no-exposure ($T_{i}=0$). The PO framework correspondingly defines the potential outcomes under-exposure and no-exposure as ${Y_{i}}^{\left( 1 \right)}$ and ${Y_{i}}^{\left( 0 \right)}$. Causal studies typically focus on average treatment effects (ATEs), which quantify the difference in mean outcomes between exposure and no-exposure groups, i.e., ${{ATE=E[Y}_{i}}^{\left( 1 \right)}]-{{E[Y}_{i}}^{\left( 0 \right)}]$, to measure the impact of an intervention on a population. For example, in neurosurgery, researchers may investigate the effectiveness of a new surgical technique$\left( T_{i}=1 \right)$compared with the current standard care ($T_{i}=0$) for treating a specific condition. A positive ATE indicates that, on average, patients receiving the new surgical technique will have better outcomes than those receiving standard care.

However, in real-world studies, researchers can only observe one potential outcome for each participant, necessitating additional assumptions to estimate the ATE from available data, as detailed in Table 2. Based on these assumptions, the ATE can be derived as

$${{ATE=E[Y}_{i}}^{\left( 1 \right)}]-{{E[Y}_{i}}^{\left( 0 \right)}] = {{E[Y}_{i}}^{\left( 1 \right)}|T_{i}=1]-{{E[Y}_{i}}^{\left( 0 \right)}|T_{i}=0]$$

$=E\left[ Y_{i} | T_{i}=1 \right]-E\left[ Y_{i} | T_{i}=0 \right]$.

The equality in the first row assumes exchangeability, meaning the treatment assignment does not depend on potential outcomes. In other words, given any other variables we've accounted for (e.g., age, sex, health status), the group receiving treatment is similar to the group not receiving the treatment in all aspects that affect the outcome. The equality in the second row requires consistency, meaning that an individual's observed outcome $Y_{i}$ under their received treatment $T_{i}$ is equal to their potential outcome under that treatment. For instance, if a patient takes the treatment and recovers, consistency assumes that this recovery is the direct result of the treatment.

Based on the aforementioned equation, the ATE can be estimated by the difference in outcome means among the exposure and non-exposure groups in the observed dataset, which can be expressed mathematically as:

$$\hat{ATE}=\frac{\sum_{i}^{N} T_{i}Y_{i}}{\sum_{i}^{N} T_{i}}-\frac{\sum_{i}^{N} \left( 1-T_{i} \right)Y_{i}}{\sum_{i}^{N} \left( 1-T_{i} \right)}.$$

- 1. **The ATE under confounding and selection bias**

In RCTs, the randomized assignment of treatments can ensure the exchangeability assumption in Table 2. However, in observational studies, this exchangeability assumption is highly likely to be violated, due to confounding and selection bias.

Confounding, a significant source of bias in observational studies, violates the exchangeability assumption and leads to inaccurate ATE estimates. In the aforementioned example, let binary variable $L_{1i}$ denote disease severity, where participants with less severe conditions ($L_{1i}=0)$ are more likely to receive the new surgical technique and tend to have a better outcome than those with more serious conditions $(L_{1i}=1)$. This violates the exchangeability assumption, as the distribution of the outcome-related variable $L_{1i}$ differs between the treatment and non-treatment groups. Consequently, $L_{1i}$ acts as a common cause (i.e., *confounder*) for the treatment and the outcome, undermining the causal effects of $T_{i}$ on $Y_{i}$ if the variable $L_{1i}$ is not conditioned on. Nevertheless, by satisfying the *conditional exchangeability assumption*, that is, the exchangeability assumption holds given the measured confounding variables, causal relationships between $T_{i}$ and $Y_{i}$ can be established within each level of $L_{1i}$. Based on the conditional exchangeability assumption, various confounder adjustment methods, detailed in Section 4, can be employed to retrieve causal relationships from observational studies in the presence of measured confounders.

Selection bias, which can arise from factors like different probabilities of loss to follow-up, nonresponse bias, or censoring bias, can also affect ATE estimates. To illustrate this, let us continue with the neurosurgical example by considering the situation that the new surgical technique ($T_{i}=1)$ can reduce mortality (denoted by $C_{i})$, and higher values of $Y_{i}$ also lead to a lower probability of mortality. In this scenario, the mortality indicator $C_{i}$acts as the common effect (i.e., *collider*) of $T_{i}$ and $Y_{i}$. If the observational study is restricted to participants who are alive (i.e., estimating the ATE conditioning on the mortality $C_{i}$), it may lead to selection bias, distorting the observed causal effects. Intuitively, participants in the non-exposure group may exabit higher values of $Y_{i}$ even if there are no causal effects of $T_{i}$ on $Y_{i}$.

- 1. **Directed Acyclic Graphs (DAG) for identifying potential bias**

So far, we have considered simplified scenarios: one single confounder and/or collider; however, the actual relationship between the exposure and outcome of interest can be more intricate, involving multiple confounders and colliders. To establish causal effects in the presence of complex relationships, a graphical tool named *Directed Acyclic Graphs (DAG)* was developed to depict the relationships between variables based on prior knowledge and assumptions. "Directed" indicates that arrows represent causation in one direction, while "Acyclic" means no circular relationships exist. The common causes of every pair of variables in the graph should be included in a DAG. By utilizing a DAG, investigators can identify the potential confounders and colliders and ascertain which variables require adjustment.

Figure 1 depicts a DAG with seven variables. The marginal dependence between $W_{3}$ and $Y$ is due to the common cause $W_{1}$, but they are conditionally independent when adjusting for $W_{1}$. Adjustments must be made for the confounding variables $L_{1}$ and $L_{2}$ to explore the causal effect of $T$ on $Y$. In this DAG, no collider is present. It should be noted that DAG does not serve as a ground truth model; rather, it reflects the investigators’ expert knowledge and assumptions regarding the causal network. These assumptions in turn can guide the actions required to obtain valid causal estimates.
